# Fragment Linker Prediction Using the Deep Encoder-Decoder Network for PROTACs Drug Design

**DOI:** 10.1021/acs.jcim.2c01287

**Published:** 2023-05-08

**Authors:** Chien-Ting Kao, Chieh-Te Lin, Cheng-Li Chou, Chu-Chung Lin

**Affiliations:** † AnHorn Medicines Co., Ltd., Taipei 115202, Taiwan; ‡ Department of Biomedical Engineering, University of California Davis, Davis, California 95616, United States

## Abstract

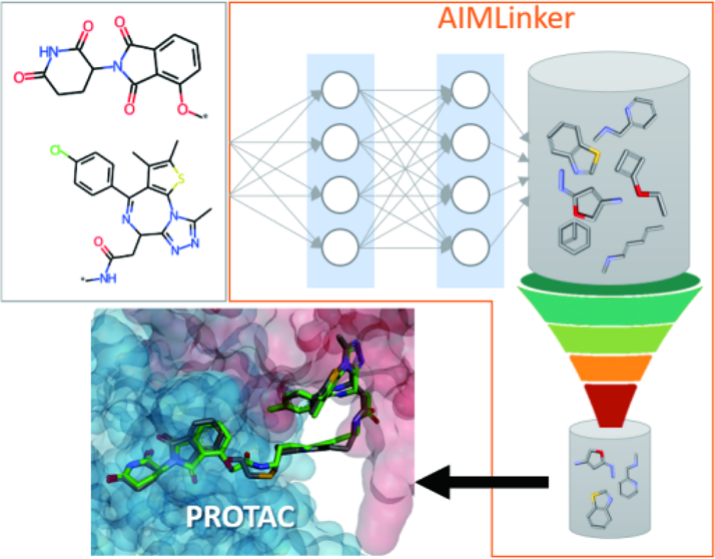

A drug discovery and development pipeline is a prolonged and complex process that remains challenging for both computational methods and medicinal chemists and has not been able to be resolved using computational methods. Deep learning has been utilized in various fields and achieved tremendous success in designing novel molecules in the pharmaceutical industry. Herein, we use state-of-the-art techniques to propose a deep neural network, AIMLinker, to rapidly design and generate meaningful drug-like proteolysis targeting chimeras (PROTACs) analogs. The model extracts the structural information from the input fragments and generates linkers to incorporate them. We integrate filters in the model to exclude nondruggable structures guided via protein–protein complexes while retaining molecules with potent chemical properties. The novel PROTACs subsequently pass through molecular docking, taking root-mean-square deviation (RMSD), relative Gibbs free energy (*ΔΔG*_*binding*_), molecular dynamics (MD) simulation, and free energy perturbation (FEP) calculations as the measurement criteria for testing the robustness and feasibility of the model. The generated novel PROTACs molecules possess similar structural information with superior binding affinity to the binding pockets compared to the existing CRBN-dBET6-BRD4 ternary complexes. We demonstrate the effectiveness of the methodology of leveraging AIMLinker to design novel compounds for PROTACs molecules exhibiting better chemical properties compared to the dBET6 crystal pose.

## Introduction

Drug design is an iterative process involving binding affinities, pharmacokinetics, and molecular structures that undergo multiple cycles before optimizing a lead drug for trials.^1^ Structure-based drug design remains challenging owing to the search space and the chemical synthesis of logical drug-like molecules.^2^ Kick et al.^3^ demonstrate the ability to couple the complementary methods of combinatorial chemistry methods and structure-based design within a nanomolar range. The structure-based design also directs the discovery of a drug lead, which is not a drug product but a compound with higher nanomolar affinity for a target.^4^ Considering the current needs and limitations of drug discovery, the demand for expanding the structure-based drug design into various targets is increasing.

Proteolysis-targeting chimeras (PROTACs) have recently drawn considerable attention to modalities. PROTACs are heterobifunctional small molecules connecting a ligand for recruiting a target protein of interest (POI) and a ligand for a ubiquitin–protein ligase (E3), with an appropriate linker that degrades a target protein.^5,6^ Degradation is initiated when PROTACs promote the POI and E3 to form a ternary complex.^7^ From a structural drug discovery point of view, the design of PROTACs is based on identifying the best combinations of three different chemical moieties as well as requires an attentive study of the structural characteristics of the E3 ligase and the POI-complemented molecular modeling and dynamics.^8,9^

Multiple E3 ubiquitin ligases have been targeted for PROTACs development and represent promising chemical properties in drug discovery. Herein, we focus on the CUL4-RBX1-DDB1-CRBN E3 ubiquitin ligase, comprising Cullin-4 (CUL4), the RING-finger protein box1 (RBX1), the adapter damage-specific DNA binding protein 1 (DDB1), and cereblon (CRBN) to form a macromolecular complex.^10^ The substrate receptor cereblon (CRL4^CRBN^) binding to immunomodulatory drugs (IMiDs) may induce cancer therapeutical effects via targeting key neosubstrates to degrade.^11,12^ The PROTACs recruiting E3 ubiquitin ligase and POI principle have been successfully applied to BRD4, a bromodomain and extra terminal (BET) family member acknowledged in cancer for its role in organizing superenhancers and regulating oncogenes’ expression.^13^ Winter et al.^14^ designed dBET6, a hybrid compound that drives the selective proteasomal degradation of BRD4 by linking to BET proteins and the CRL4^CRBN^ ligand (hereafter called CRBN).^15^ The chemical properties, such as molecular weight, polar surface area, number of H-bond acceptors, and number of H-bond donors, have been proven to affect the structural rigidity, hydrophobicity, and solubility of PROTACs molecules.^16,17^ Research has been conducted on rational PROTACs design through structural biological and computational studies, but linker design and generation remain unclear.

Recent studies have leveraged the aid of rapid simulation and state-of-the-art deep learning to discover novel structures, demonstrating the feasibility of timely and accurate screening of potential targets.^18,19^ graph neural network (GNN) is one of the techniques gaining considerable attention in drug discovery because it automatically learns task-specific representations using graph convolutions and conserves the graph information as atom-bond interactions.^20,21^ GNN learns the representations of each atom by aggregating the information from its surrounding atoms that is encoded via the atom feature vector and recursively encodes the connected bond feature vector through the message passing across the molecular graph, followed by a readout operation that forms corresponding atoms and bonds.^22−24^ The modern GNN models in predicting properties have proven to be superior or comparable to traditional descriptor-based models.^25,26^ Wu et al.^27^ showed the evaluated results that GNN outperformed on most data sets, giving the network the feasibility of predicting various chemical end points. Thus, GNN has been proven to be a potential model for designing and generating novel structures for drug discovery and investigating drug-like candidates.

Further, a gated graph neural network (GGNN) outperforms molecular graph generation in deep generative models^28,29^ and demonstrates the practical structure formation in drug design.^20,30^ Many approaches use two-dimensional (2D) SMILES-based chemical graphs embedded in low-dimensional space to generate new molecules by perturbing the hidden values of the sampled atoms.^31−34^ These studies are missing the nature of molecular shape and the three-dimensional (3D) information, which may considerably differ from the starting point of structure design. Another recent popular deep neural network drug design is in the fragment linking technique. DeLinker,^35^ adapted from Liu et al.,^32^ is the first attempt to apply GNN in linker design, particularly retaining the 3D structural information and generating linkers by giving two input fragments. 3DLinker^36^ predicts the fragment nodes and sampling linker molecules simultaneously. However, none of these studies have demonstrated an effective method for refining the generated molecule nor further considered validation in molecular conformations. This study still lacks an integration pipeline for adapting deep neural networks as the core technique in drug discovery and substantial validation process are still lacking in investigation.

This study proposes a data-driven deep learning-based neural network methodology, artificial intelligent molecule linker (AIMLinker). This network integrates designing, generating, and screening novel small molecular structures for PROTACs linkers, demonstrating a highly effective methodology for creating neostructures to address the current difficulties in drug discovery. AIMLinker considers the structural 3D information that initially takes two fragments with predefined anchors on both sides and their structural information on angle and distance to represent the spatial positions between the input fragments. The core architecture of the network is GGNN^37^ with atoms and bonds represented as nodes and edges, respectively. In addition, the iterative process of adding atoms and forming bonds is repeated until termination, followed by a readout step for returning a newly generated compound and subsequently screening with the postprocess step. The generated molecules are docked back to the CRBN-BRD4 complex through AutoDock4 and validated by measuring the root-mean-square deviation (RMSD), the relative Gibbs free energy (*ΔΔG*_*binding*_), molecular dynamics (MD) simulation, and free energy perturbation (FEP) simulation to test the robustness and feasibility as a drug-like molecule. This end-to-end pipeline demonstrates a novel method for using state-of-the-art deep learning techniques for drug discovery and shows the viability of designing novel PROTACs linker molecules.

## Methods

**Figure 1:**
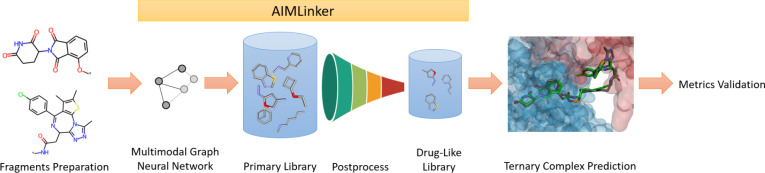
Scheme of the pipeline. The starting input data is two fragments, which are preprocessed and have their relative structural information. Next, AIMLinker takes the input fragments and generates the linker molecules. Finally, the molecules are postprocessed with our algorithm. We then take the best docking results of four molecules to validate their robustness of recognition as drug-like molecules.

In this section, we first provide the details on preprocessing the POI and E3 ubiquitin ligase structures selected as the input for our encoder-decoder network. Next, we present the network architecture to generate the linker molecule with good viability and reasonable for drug synthesis. Next, the postprocessing procedures are provided for validating the predicted molecules and conserving drug-like PROTACs molecules. Finally, the robustness of our predicted molecules is evaluated via docking and binding affinity metrics. The overall pipeline of our study is shown in Figure 1.

### Data Processing

**Figure 2:**
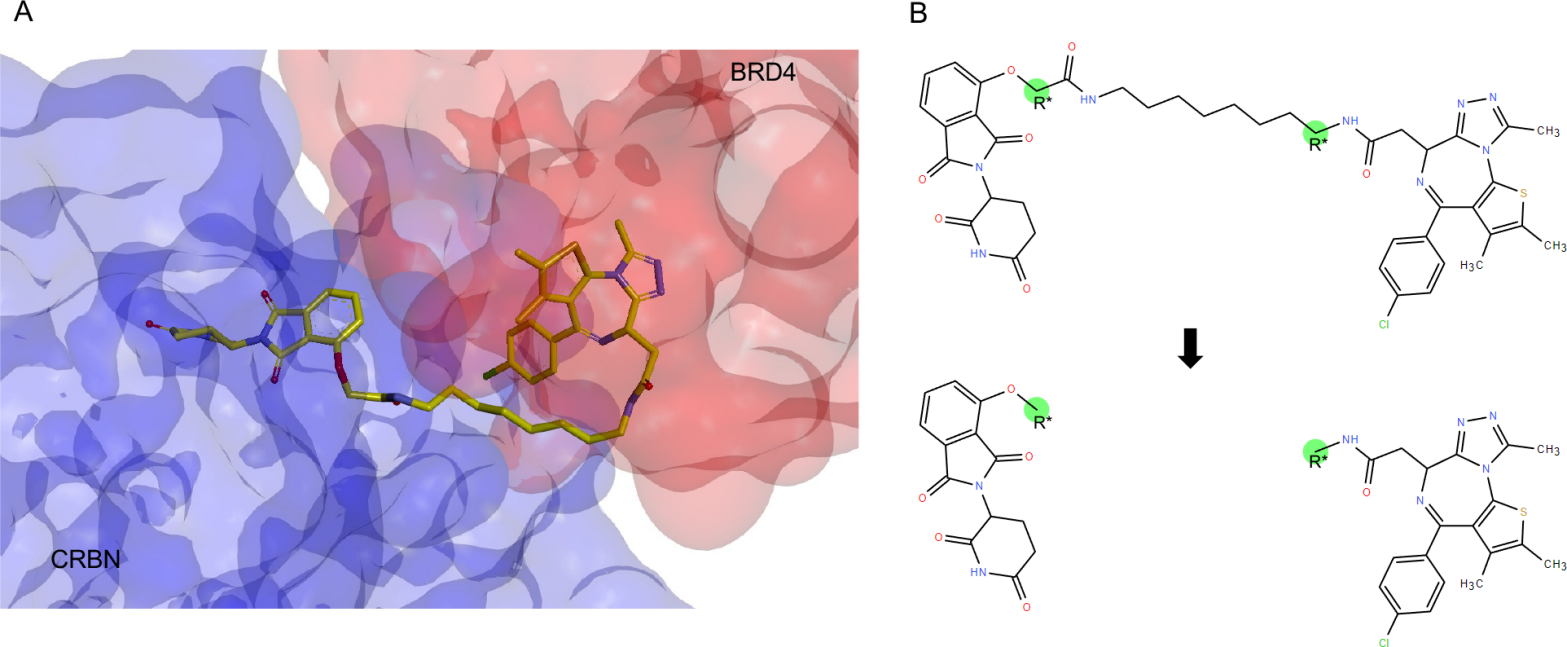
Scheme of the CRBN-dBET6-BRD4 ternary structure and processing protocol of dBET6. (A) The structure and spatial information on 6BOY with the linker binding to BRD4 and CRBN. The red and blue labeled proteins represent BRD4 and CRBN, respectively. (B) 2D illustration of the dBET6 molecule links to E3 ubiquitin ligase and POI ligase. First, the anchors are highlighted and labeled with R* to feed the network with the start and end positions of the generated linkers. Next, the molecule between the anchors is removed, and the remaining two fragments are considered the input data for the network.

The plastic binding between the ligase and the substrate adopts distinct conformations depending on the linker length and position. dBET6, a PROTACs molecule, exhibits high selectivity properties with the structure, and Nowak et al.^38^ provided the ternary cocrystal structure of CRBN-dBET6-BRD4 (PDB: 6BOY) in the Protein Data Bank.^39^ The integration of structural, biochemical, and cellular properties of the 6BOY ternary complex is designed to be a neodegrader-mediated PROTACs structure. Figure 2A illustrates the relative spatial pose of the CRBN-dBET6-BRD4 ternary complex via Discovery Studio Visualizer (DSV),^40^ where the red-labeled and blue-labeled structures are BRD4 and CRBN, respectively. dBET6 is the bridging molecule to link E3 ubiquitin ligase CRBN and target protein ligase BRD4.

The mechanism of PROTACs forces the target protein to dock to the E3 protein, so that the two proteins and PROTACs form a ternary complex, regardless of the nature of the two undockable proteins. In data preparation, we take the docking pose of the CRBN-BRD4 complex and the corresponding binding moieties to design the PROTACs linker. Therefore, the input data for the network is two fragments comprising two ligands extracted from the PROTACs excluding the linker moiety. We first retrieve the PROTACs molecule from the 6BOY structure to prepare the input data from dBET6. Considering the potent BRD4 inhibitor examined by Filippakopoulos et al.,^41^ the fragment of the BRD4 ligand is defined as an illustration in Figure 2B, while the CRBN ligand is defined as a pomalidomide-like structure. The linking anchors on each ligand are labeled with R*, and the linker between these two anchors is removed. The anchors provide the 3D spatial information on the angle and distance between the two fragments because the cocrystal structure retrieved from the PDB is spatially predefined and fixed. The network further takes the two fragments and the corresponding spatial information as the input to generate and design a linker library with the constraint of the space between the anchors.

### Multimodal Encoder-Decoder Network

We propose a data-driven deep learning network, AIMLinker, integrating the generation and design of novel structural linkers between input fragments and postprocessing the predicted structures. This network is developed based on the reports by Imrie et al.^35^ and Liu et al.^32^ To develop the network, two unlinked fragments with information regarding relative spatial position and orientation were used to generate the linker structures that bound to the anchors on both fragments, forming a novel molecular structure. In the network, we created a new training data set focused on reconstructing PROTACs linker molecules and trained and fine-tuned the network to address the unmet needs for designing such linkers. In addition, we used postprocess filters to refine the generated molecules and retain the most potential drug-like molecules. The architecture of the network is inspired by the reports of Imrie et al. and Liu et al. However, we modified the network to train and fine-tune the AIMLinker to meet our needs for producing PROTACs molecules.

The generation process is achieved via iteratively generating edges and adding new atoms from the selected pool, specifically 14 permitted atom types. The model generates the molecules in a breadth-first manner with a masking step to apply basic atomic valency rules. In addition, the network allows users to define the number of atoms between the anchors to maximize the variations in generating the new linker molecules and provide the validity size of the two fragments corresponding to their distances. The other selection is the number of molecules to be generated, and the network includes a postprocessing step to remove the molecules not subject to basic chemistry rules, duplicates, and illogical structures.

**Figure 3:**
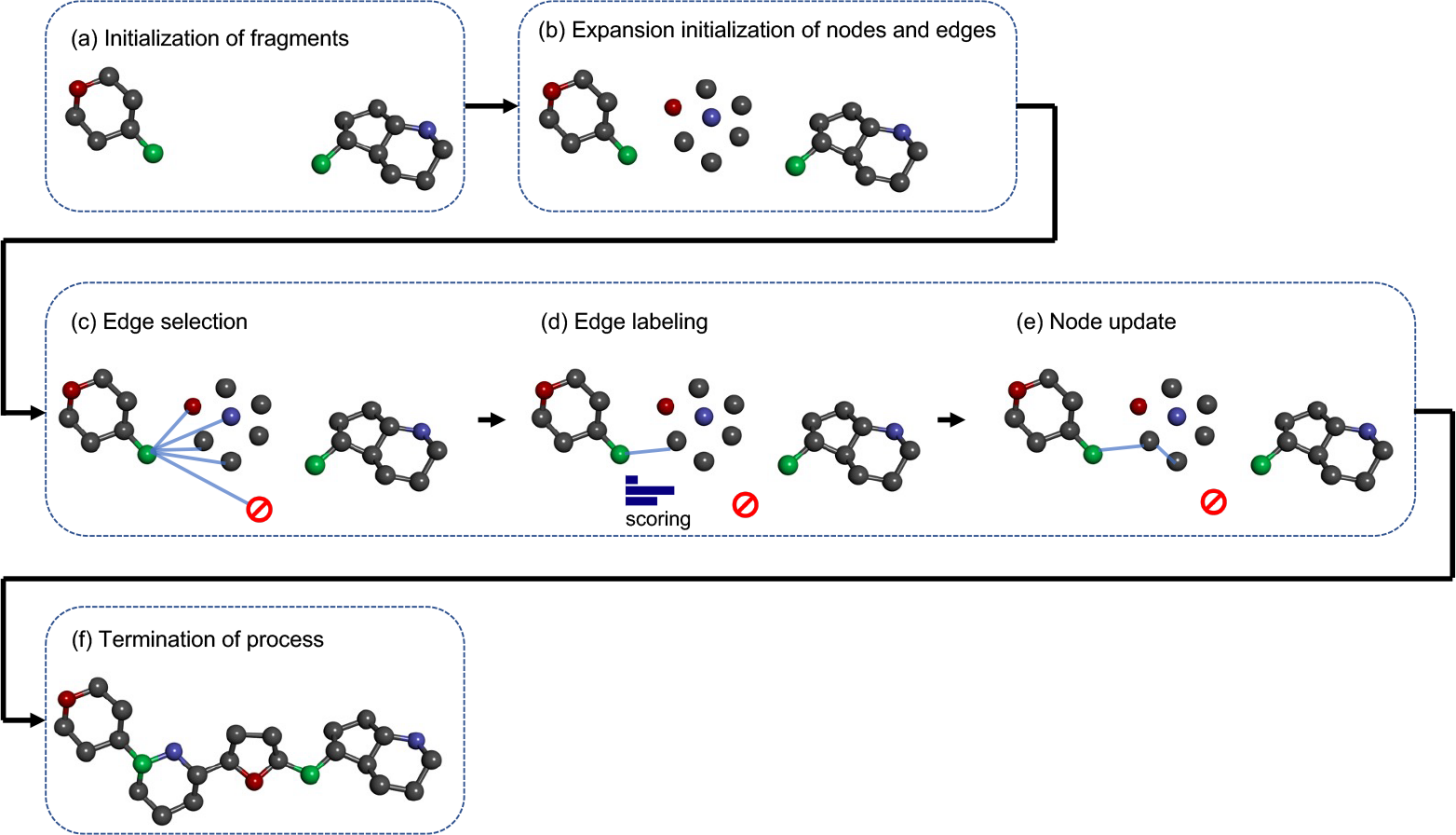
Network generation process. Two fragments in (a) are generated with the data processing steps, providing the spatial information, and the angle and distance between the anchors are calculated accordingly. Initialization of the nodes and edges in the network is illustrated in (b), where the 14 permitted atoms are randomly selected between the space. From (c) to (e) are the steps to process edge selection, edge labeling, and node updates. In particular, the three steps are sequentially repeated operations until the atom number reaches the maximum setting or all the edges and nodes are generated to cause (f) termination of the process.

Figure 3 illustrates the iteration process, where the network uses a standard GGNN. The input fragments prepared from the data processing step are turned into a graph representation. Each atom and bond represent node and edge and are labeled as *z* and *l*, respectively. A list of allowed 14 atom types is provided in the Supporting Information. As shown in Figure 3a, the graph information is passed through AIMLinker, which utilizes GGNN as the core encoder structure, and the hidden state of nodes and edges is updated to integrate during the learning process (Figure 3a).

Next, the graph representation of fragment input initializes with node expansion, as shown in Figure 3b. Each node has a random hidden state *z*_*v*_ derived from the *d*-dimensional normal distribution of , where *d* represents the number of features of the hidden state. The expansion nodes are subsequently labeled as *l*_*v*_ association with structural information sampled from the SoftMax output of a learned mapping *f*. The function *f* is applied with a linear classifier. However, it can be substituted with other functions to map the hidden state *z*_*v*_ into different atom types *l*_*v*_. Notably, the selected linker length can limit the number of expansion nodes.

Figure 3c–e shows that iteratively selecting edges, labeling edges, and updating nodes generate new molecules. First, the initial node *v* considers whether to form an edge to the neighborhood node *u* in the graph. It is selected when the queue starts, which is the initial input fragment anchors configure. The node is added to the queue if it is first connected to the graph. These processes are repeated until the expansion nodes are all queued, and no further nodes can be formed (i.e., termination of process in Figure 3f). The edge of the nodes *v* and *u* considers the basic valency constraint. The *f*, representing the core network GGNN, constructs a feature vector with the subsequent node *u* with such *l*_*u*_ ∼ *f*(*z*_*v*_^*t*^). The edge between *v* and *u* at time point *t* is considered via a feature vector ϕ_*v*,*u*_^*t*^
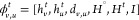
where *h*_*v*_^*t*^ and *h*_*u*_^*t*^ represent the hidden state of the initial node *v* and subsequent *u*, respectively. *d*_*v*,*u*_ indicates the distance between the two nodes in the graph. *H*^0^ is the local information on the nodes, while *H*^*t*^ shows the global information on the nodes at the time point *t. I* is encoded with 3D structural information on the relative angle and distance of the input fragments. The following representation is used to produce a distribution over the candidate edge:
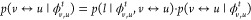
The edge between the two nodes *v* and *u* is formed in single, double, or triple bonds after *u* is selected. Notably, bond formation is subject to basic valency constraints.

Finally, all the nodes are updated via their hidden state in accordance with the neural network in Figure 3e. We calculate the new hidden state  from the initial hidden state  with considering the neighborhood nodes. At time point *t* + 1, we discard the previously hidden state  and conserve the local graph information on the queued nodes. This process suggests that molecule generation is independent of the graph history and solely considers the local graph information. The iteration process of Figure 3c–e will terminate when all the queues are empty. At the end of the generation, the largest intact molecule will be returned (Figure 3f), and the unconnected nodes will be discarded. The stereochemistry information on the generated molecules is not given during the generative process. A postprocessing step is needed to screen the predicted molecules and test their robustness.

### Model Training

We prepared a conventional ZINC data set^42,43^ and PROTAC-DB,^44^ to train under a variational autoencoder (VAE) framework. We constructed the training data set of 160,491 molecules with 157,221 and 3,270 from ZINC and PROTAC-DB, respectively. The chemical compounds with heavier and more complex structures in the ZINC data set were selected. Meanwhile, in the PROTAC-DB data set, all the compounds to date are selected. Each compound is segmented into two fragments and one linker as the input for the network. We construct the linker segment to contain at least three heavy atoms while retaining the intact ring structures in the linker or the two fragments. Next, we split the data set into 90% for training and 10% for the validation process by adapting a 10-fold cross-validation to overcome the overfitting issue. Finally, we tune the hyperparameter as reflected in Table S1 to retrieve the best performance.

The model trains on the data set focusing on the fragment-molecule pairs. Given the two input fragments X and linked molecule Y, the goal of the model is to reconstruct Y from (X, *z*). The original linked molecule Y is transferred into a graph representation, and *z* is the latent code of the learned mapping. Specifically, *z* is learned from a set of expansion nodes of Y, and the mapping is averaged into a low-dimensional vector. We constrain *z* into the low-dimensional vector to enforce the network learning the information from Y and then degenerate the network for Y. The loss function  between encoder bias θ and decoder bias φ is similar to standard VAE loss, including a reconstruction term and a Kullback–Leibler regularization loss
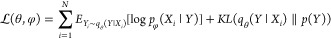
where the first term of reconstruction loss represents the prediction of the atom types *l*_*v*_ and encourages the model to learn to reconstruct the data producing the target molecule. We denote the encoder as *q*_θ_(*Y* | *X*), which is a Gaussian probability density of the *i*-th node position. The decoder is denoted by *p*_φ_(*X* | *Y*) to reconstruct the input molecule Y from X. The second term of Kullback–Leibler regularization diverges the error distribution between the predicted molecule spatial distribution *q*_θ_(*Y* | *X*_*i*_) and the probability vector *p*(*z*), which is derived from the linked molecule, B. Notably, we allow variations from the pure VAE loss, where the concept was introduced by Yeung et al.^45^

### Postprocessing

**Figure 4:**
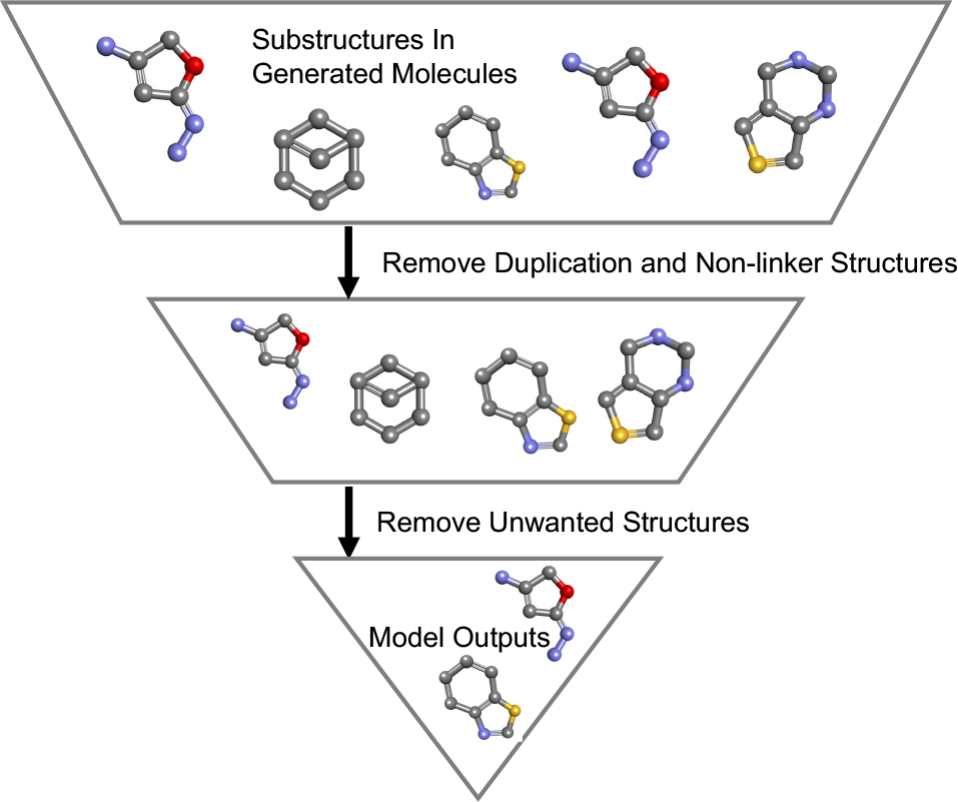
Workflow of postprocess. The generated molecules pass through multiple steps of filters, specifically removing duplicates, nonlinker, and unwanted substructures. This postprocess step is integrated into AIMLinker.

The raw outputs from the model are PROTACs library in the 2D chemical structure and are further postprocessed with our screening procedures to remove the unfavored targets. Figure 4 shows the filters of our proposed method that are integrated into AIMLinker. Owing to the constraint of the graph computational process and the linked substructures, the model generates the molecules by choice, including duplicated predictions, undruggable targets, and structures violating the basic chemistry law. The first filter eliminates the duplicates, which are the same structures predicted by the model, leaving every unique molecule after this process. This process also includes the nonlinker substructures, i.e., two fragments are not formed into one compound with the linker moiety. Next, we have a library with unfavorable substructures that are not feasible for chemical synthesis or cannot become a druggable target. This library includes the substructures such as acid halide, disulfide bond, peroxide bond, and small-number cyclic rings with double bonds and additionally the model to predict whether the newly generated substructures are feasible as a drug lead. This step is important for screening the target pool to reduce the number of molecules while retaining the candidates potentially having high binding affinity and chemical activity. Finally, the molecules that violate Bredt’s Rule,^46^ in which the substructures contain certain bicycle atomic bridged-ring structures with a carbon–carbon double bond at a bridgehead atom, are removed from the target pool. These steps remove the unwanted molecules from the target pool, and the remaining targets considerably reduce the needed computational resources and the time span for simulation.

We utilize the “Rule of Three” to measure the effectiveness of our generated molecules and to further validate the merit of using postprocess steps. “Rule of Three” refers to the molecular weight (MW) of a fragment being < 300 Da, the calculated logarithm of the 1-octanol–water partition coefficient of the nonionized molecule (cLogP) being ≤ 3, the number of hydrogen bond donors (HBDs) being ≤ 3, and the number of hydrogen bond acceptors (HBAs) being ≤ 3. Further, we also include the polar surface area (PSA) of being ≤ 60 Å in addition to the standard setting of the rule.^47,48^ We apply this rule to the generated linker pool to measure and calculate the molecular properties at each filter step.

### Docking Validations

The 3D protein–protein interaction poses and 3D conformations of postprocessed molecules need to be constructed first before applying the docking methods. The cocrystal structure of CRBN-dBET6-BRD4 is released in the PDB and can retrieve the simulated spatial conformation via DSV. Meanwhile, all postprocessed molecules, initially sketched as 2D chemical structures, are converted into 3D PROTACs conformations through DSV. The reference compound dBET6 is also reconstructed into a series of 3D conformations to validate the consistency of our docking methodology. These 3D compounds are subsequently minimized using the energy minimization method.^49^ After protein–protein interaction poses and 3D conformations of PROTACs candidates are well prepared, AutoDock4^50^ is applied to predict the best PROTACs binding pose by labeling the binding pocket as a grid. Each 3D PROTACs freely binds to CRBN and BRD4 with consideration of the binding energy, biochemistry property, and entropy during the docking procedure. We allow 10 binding poses of each PROTACs from the network and form a 10-time data set as the initial docking inputs. Therefore, our PROTACs library and dBET6 can freely rotate, fold, and bind to the pocket to form the best pose in DSV with the highest binding affinity and lowest entropy energy.

We measured the metrics, including structural information and binding affinity, to validate the robustness of the generated molecules from AIMLinker and dBET6 provided in the PDB. We use the RMSD, which was introduced by Bell et al.^51^ for quantitatively measuring the similarity between respective atoms in two molecules
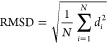
where *N* is the number of atoms in the ligand, and *d*_*i*_ is the Euclidean distance between the *i*^*th*^ pair of corresponding atoms. We take the crystal structure in the PDB as the reference compound and measure the structural similarity level with the generated linker molecules by superimposing and calculating the RMSD values.

Another metric considered for validating the molecules is *ΔG*_*binding*_ of the protein–ligand complex. It is calculated from the molecular mechanics Poisson–Boltzmann surface area (MM-PBSA) method.^52^ The MM-PBSA approach is one of the most widely used methods to compute the interaction energies among biomolecular complexes. In general, *ΔG*_*binding*_ between a protein and a ligand in a solvent can be expressed as
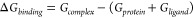
where *G*_*complex*_ is the total free energy of the protein–ligand complex, and *G*_*protein*_ and *G*_*ligand*_ are the total free energies of the separated protein and ligand in the solvent, respectively. We individually generate 10, 25, 50, and 100 poses of each molecule, and AutoDock4 determines the best logical spatial orientation of each trial independently. The best pose of each trail is retrieved and applied to the docking process to calculate the *ΔG*_*binding*_. The metric is averaged between trials with variations. Finally, we constrain the CRBN-BRD4 spatial position and consider the free movement of the generated bridging molecules.

### Molecular Dynamics Simulation

We collected the best linker structure to compare its *ΔG*_*binding*_ and *ΔΔG*_*binding*_ with redocked dBET6 and further examine the robustness of the generated molecule. The binding affinities of the three selected PROTACs (dBET6 crystal pose, dBET6 redocked pose, and 6BOY_1268) with CRBN-BRD4 are further evaluated with 10 ns MD simulations via GROningen MAchine for Chemical Simulations (GROMACS) 2022.4 version.^53^ Each CRBN-PROTACs-BRD4 ternary complex was constrained using the CHARMm force field^54^ and solvated using the transferable intermolecular potential with three points (TIP3P) water molecules in a truncated octahedron water box with a minimal distance of at least 10 Å from any edge of the box to any protein atom. Each CRBN-PROTACs-BRD4 complex energy was initially energy minimized to remove the unfavorable contacts using the conjugate gradient method for 10,000 steps and subjected to 100 ps of heating from 50 to 300 K. Subsequently, a 500 ps equilibrium run was performed. Finally, periodic boundary dynamics simulations of 10 ns were conducted for the production step in an NPT ensemble at 1 atm and 300 K. All covalent bonds involving hydrogen were constrained during the simulations using the SHAKE algorithm. The particle mesh Ewald method was used to treat the long-range electrostatic interactions.^55^ The output trajectory files were saved every 2 ps from a 10 ns period and used for subsequent binding free energy analysis.

To calculate binding free energy *ΔG*_*binding*_, the 5,000 frames from the 10 ns trajectory of CRBN-dBET6-(crystal pose)-BRD4, CRBN-dBET6-(redocked pose)-BRD4, and the CRBN-6boy_1268-BRD4 ternary complex are individually calculated and averaged by applying the MM-PBSA method. The relative binding free energy *ΔΔG*_*binding*_ is measured by comparing the dBET6 crystal pose with redocked dBTE6 and 6boy_1268, respectively.

## Free Energy Perturbation Simulation

We adopt the FEP simulation to measure the generated relative binding energy of the generated molecules. This calculation is useful for predicting ligand–protein binding affinities via molecular simulations and measuring the phase transition. The three selected PROTACs (dBET6 crystal pose, dBET6 redocked pose, and 6boy_1268) with CRBN-BRD4 were evaluated using FEP within nanoscale MD (NAMD).^56,57^ We use 20 lambda windows (0.0, 0.01, 0.05, 0.1, 0.2, 0.3, 0.4, 0.5, 0.6, 0.7, 0.8, 0.85, 0.9, 0.91, 0.93, 0.95, 0.96, 0.97, 0.98, 0.99, and 1.0) in the FEP schedule run for 8 ns and conduct three independent FEP schedule runs. The relative binding free energy between two ligands (L0 to L1, *ΔΔG*_*binding*_^L0→L1^) is defined as
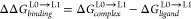
where *ΔG*_*complex*_^L0→L1^ is the free energy change upon transforming L0 to L1 in the complex, and *ΔG*_*ligand*_^L0→L1^ is the energy change in the solution.

## Results and Discussion

We developed a data-driven network, AIMLinker, generating the neostructure of a small molecule linker to PROTACs degradation protein. AIMLinker takes two fragments with structural information as the input data and processes a deep learning network to create linker molecules. First, we provide the details of the generated molecules with their chemical properties and structural statistics. Second, we take four molecules with the highest binding affinity to compare dBET6 with their RMSD and *ΔG*_*binding*_. Finally, the generated molecule with the best chemical properties is substantially taken to verify via MD simulation and FEP simulation.

AIMLinker demonstrates a robust and rapid pipeline for generating and designing new PROTACs linkers. Our network combines two processes into one end-to-end pipeline: It 1) takes two unlinked fragments as input and uses an encoder-decoder deep learning network to generate the substructures forming a new PROTACs molecule [We considered the structural information in the form of the angle and distance between the two initial fragments and iteratively added atoms between the space until it was filled or the limitation of maximum atom setting was reached.] and 2) postprocesses the generated molecules to extract the potential drug-like molecules. We screen for duplicates and exclude structures violating basic chemistry rules and unwanted substructures. This rapid pipeline allows the viability of timely generating novel small molecules with high binding affinity to CRBN-BRD4 and the potential to translate the work to other PROTACs targets.

### Generated Molecules

We generated the molecules with a specific range of fragments. This range gives flexibility to the network in designing a more linear or ring-like structure. The raw output from the neural network is 2,000 structures, including illogical molecules and unwanted substructures. Therefore, we take these outputs and subject them to postprocess procedures with two filters applied: 1) the first filter removes the duplicated molecules and nonlinker structure, i.e., two fragments do not combine to form one compound using the linker structure. After this filter, the remaining number of molecules is 1,175. The remaining molecules in this filter are unique and novel but not favorable to become a drug leads, and 2) the unwanted substructures that are not applicable to drug-like molecules are filtered out. The final output number from the AIMLinker is 524 molecules. Our model generates new and effective molecules with a comparable success rate to other state-of-the-art ML methods shown in Table S1. We highlight the second postprocess screening approach to retain the druggable and potential drug leads.

Next, we utilize the “Rule of Three” to validate the effectiveness of applying postprocess steps in the linker structure. Table 1 shows the Rule of Three metrics of MW, cLogP, HBD, and HBA, and we additionally include PSA here. The generated pool of molecules applied with the postprocess step outperformed the preprocessing step except for cLogP. Specifically, our proposed method has 93%, 95%, 95%, 60%, and 48% of the molecules that pass the rules in MW, cLogP, HBD, HBA, and PSA, respectively. For the preprocessed molecular pool, it achieves 91%, 97%, 90%, 49%, and 36% in the corresponding metrics. In addition, this method surpasses the preprocessed data with as high as 12% in PSA, while the lowest surpassed percentage compared to the preprocessed data is 2% in MW. We perform better with this additional postprocess step in four out of five metrics, demonstrating the robustness of the linker molecules possessing better chemical properties.

**Table 1:** Results of “Rule of Three” Parameters Analysis^a^

Parameters	Preprocess (%)	Postprocess (%)
MW (<300 Da)	91	**93**
cLogP (≤3)	97	95
HBD (≤3)	90	**95**
HBA (≤3)	49	**60**
PSA (≤60 Å^2^)	36	**48**

a Abbreviations: molecular weight, MW; the calculated logarithm of the 1-octanol–water partition coefficient of the nonionized molecule, cLogP; the number of hydrogen bond donors, HBD; number of hydrogen bond acceptors, HBA; polar surface area, PSA.

Table 2 shows the structural statistics for the final output from AIMLinker. The generated molecules from AIMLinker provide ring-shape structures, while the dBET6 linker is a linear structure, giving the compound more flexibility to rotate inside the binding pockets freely and the opportunities of binding to other positions to reduce the compound potency and the pharmacokinetics property. Our generated linker structures between the two input fragments provide 229 ring-like substructures out of 524 molecules and 43% of the total number. Of the 229 compounds, 32 have bicyclic rings, and one compound has tricyclic rings. Table 2 shows the incidences of ring-shape structures of the designed molecules with different numbers of three-membered, four-membered, five-membered, and six-membered rings and different numbers of atoms in the ring structure above 6 as 24, 30, 90, 112, and 6, respectively. These cyclic compounds restrict the rotational angles and the possibility of binding to nontarget binding positions. In addition, the ring-link structures generated by AIMLinker provide more stability for the compound and possess the ability to form strong π bonds increasing the binding affinity in the binding pockets.

**Table 2:** Ring-Structure Statistics of the Generated Molecules^a^

Ring structures	Number of molecules
3-Membered ring	24
4-Membered ring	30
5-Membered ring	90
6-Membered ring	112
Above 6-membered ring	6

a The number of incidences of different numbers of membered rings.

In Table 3, we compare the performance with other existing ML methods (DeLinker and DiffLinker). The validity rate measures whether the generated molecule follows the basic chemistry law and whether the molecules are linked to the ligands. The unique rate reflects the different ratios of duplication among the valid molecules. In the novelty calculation, we extract the linker structure from the generated molecules and identify whether the linker matches the training data set. The ratio indicates the capability of the model to learn from the training data set and generate new molecules. The table shows the comparison between AIMLinker, DeLinker, and DiffLinker. AIMLinker consistently produces better results than DeLinker. This suggests the novel training data set is learned from the model and is particularly applicable to generating more robust molecules. We note that AIMLinker is inferior in validity and the novelty rate compared to DiffLinker. However, the linkers generated from DiffLinker are linked to different anchors than our initial settings. DiffLinker indeed produces novel and valid molecules, but those molecules are not correctly linked and are treated as “wrong” molecules.

**Table 3:** Performance of Generated Molecules Compared to DeLinker and DiffLinker

	AIMLinker	DeLinker	DiffLinker
Valid (%)	59.8	45.5	92.0
Unique (%)	98.3	97.1	100.0
Novel (%)	**100. 0**	99.9	100.0
Linked correct anchors (%)	**100. 0**	100.0	0

### Docking Performance

**Figure 5:**
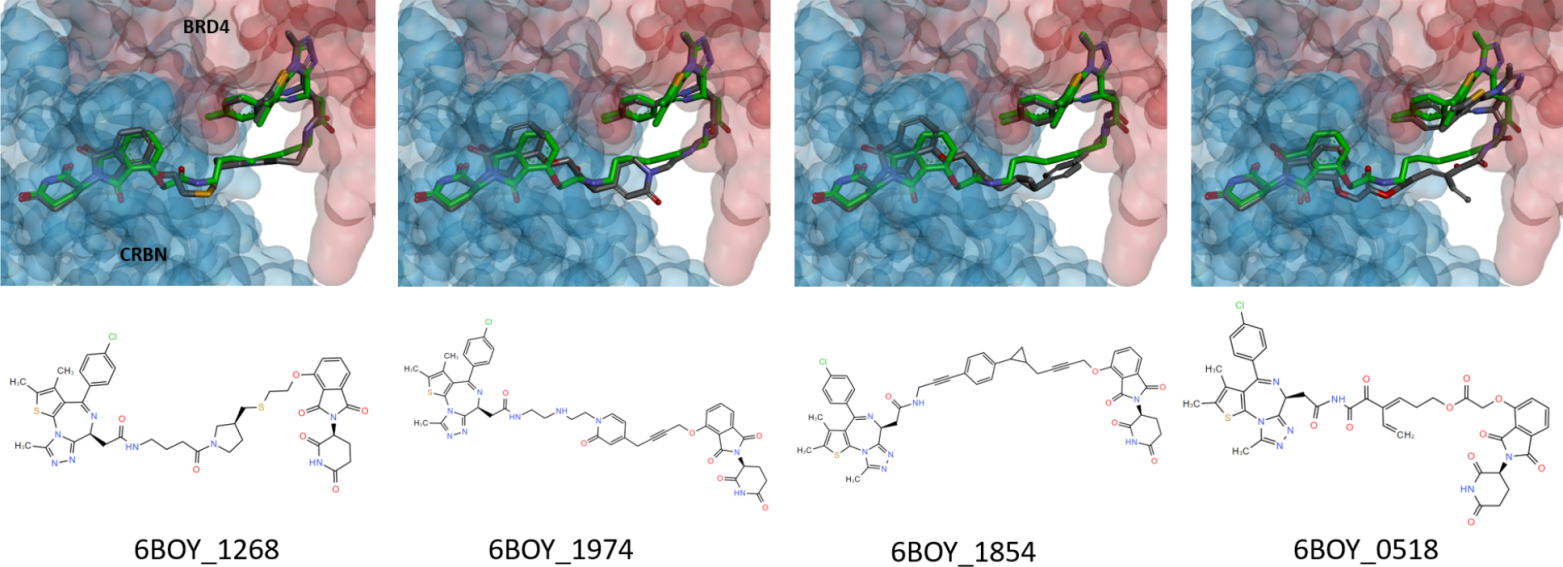
Docking poses of the generated molecules with the RMSD value < 1 Å, which takes the dBET6 conformation as the reference compound.

We use AutoDock4 for docking and validation to assess the generated molecules and to compare them with the existing dBET6 structure. We redock the compound to the binding pockets of CRBN and BRD4 to consolidate AutoDock4 having the ability to be a reference tool for measuring the generated molecules. We constrain the free energy of dBET6 to retrieve the closest docking pose and binding affinity provided in the 6BOY crystal structure. The final output of 524 molecules from AIMLinker is then passed through AutoDock4. In the standard protocol and matching with the biological interaction, we allow a maximum of 10 binding poses for each molecule. The total number generated from docking is 5,095 poses because not every molecule is feasible to bind within the pocket. We set the RMSD threshold value of ≤1 Å to be considered as drug-like molecules. The four displayed molecules in Figure 5 are extracted from this threshold.

In Table 4, we show the average RMSD and average *ΔG*_*binding*_ values. The spatial structural information is measured in RMSD values. 6BOY_1268 achieves the best average RMSD of 0.44 Å among the redocked dBET6 and four generated molecules, while 6BOY_1974 performs the second best with an average RMSD of 0.46 Å. For the average *ΔG*_*binding*_, both 6BOY_1268 and 6BOY_1974 possess better binding affinities. This performance suggests that the generated molecules have superior chemical properties than the crystal pose and redocked pose of the dBET6 molecule. Further, the third-best molecule, 6BOY_1854, exhibits comparable chemical properties to the redocked dBET6. The results demonstrate that the model generates novel and comparable molecules in PROTACs drug design.

**Table 4:** Docking Performance of Redocked dBET6 and the Generated Molecules^a^

Linker molecules	Average RMSD (Å)	Average *ΔG*_*binding*_ (kcal/mol)
dBET6 (crystal pose)	–	–22.51
dBET6 (redocked pose)	0.59 ± 0.01	–54.67 ± 11.53
6BOY_1268	**0. 44** ± **0.05**	–**62. 26** ± **6.36**
6BOY_1974	0.46 ± 0.06	–55.33 ± 4.23
6BOY_1854	0.58 ± 0.07	–56.86 ± 8.07
6BOY_0518	0.77 ± 0.08	–50.41 ± 5.23

a We individually generate 10, 25, 50, and 100 poses of each molecule and select the best pose via AutoDock4 simulation. The metrics are shown on average of the four trials with variations.

### Molecular Dynamics Simulation

Table 5 shows that the CRBN-dBET6-(crystal pose)-BRD4, CRBN-dBET6-(redocked pose)-BRD4, and CRBN-6boy_1268-BRD4 ternary complexes were subjected to 10 ns MD simulations, and the binding free energies were calculated after molecular docking. From the average *ΔG*_*binding*_ value, the complexes formed between 6boy_1268 and CRBN-BRD4 present the lowest calculated values, suggesting that 6boy_1268 forms the most stable complexes with CRBN-BRD4 compared to the values of the benchmark compound (dBET6 crystal pose and redocked pose). Further, the *ΔΔG*_*binding*_ of 6BOY_1268 is lower than the redocked dBET6 pose, indicating a better binding affinity in the CRBN-BRD4 pocket. These results further consolidate the robustness of 6BOY_1268, possessing better chemical properties than the redocked pose and potentially becoming a potent drug target.

**Table 5:** Average *ΔG*_*binding*_ and *ΔΔG*_*binding*_ Values for the Crystal Structure dBET6, Redocked dBET6, and Generated Linker with Best Chemical Properties

Compound name	*ΔG*_*binding*_ (kcal/mol)	*ΔΔG*_*binding*_ (kcal/mol)
dBET6 (crystal pose)	–45.17 ± 5.77	–
dBET6 (redocked pose)	–39.82 ± 5.02	5.35
6BOY_1268	–**45. 91** ± **4.78**	**-0.74**

## Free Energy Perturbation Simulation

We show the FEP method to calculate the relative binding affinity of protein–ligand interaction. Table 6 shows the relative binding free energy of the dBET6 crystal pose to the dBET6 redocked pose and 6boy_1268. We show the results with three independent runs and calculate the values with variations. The results demonstrate a similar finding to the previous MM-PBSA performance. The relative binding energy is stronger in linker 6BOY_1268 and CRBN-BRD4 protein complexes. Our best-generated molecule, 6BOY_1268, has higher protein–ligand binding energy, and taking the differences between the energy of the dBET6 crystal pose, the simulation shows an average of −1.25 kcal/mol. This result further supports the use of using AIMLinker to generate potential novel drug-like molecules.

**Table 6:** Relative Binding Free Energies between Pairs of BRD4 PROTACs

Compound pairs	Runs	*ΔΔG*_*binding*_ (kcal/mol)	*ΔG*_*complex*_ (kcal/mol)	*ΔG*_*ligand*_ (kcal/mol)
dBET6 (crystal pose)/dBET6 (redocked pose)				
	1	4.39	3.47	–0.92
	2	1.93	–0.66	–2.59
	3	3.21	3.49	0.28
	Average	3.18 ± 1.23		
dBET6 (crystal pose)/6BOY_1268				
	1	–0.96	1.08	2.04
	2	–2.21	–1.72	0.49
	3	–0.59	0.09	0.68
	Average	–**1.25** ± **0.85**		

## Conclusion

This study proposes a deep neural network to generate and design novel PROTACs molecules. We collectively integrate sampling and postprocessing steps to extract the potent drug-like molecules and demonstrate the robustness of the generated molecules. The generated structures possess comparable or superior chemical properties to the existing crystal structure. Furthermore, the model can perform virtual high-throughput screening for rapid generation and reduce manual labor.

Notably, the current generation process has the limitations. We take the docking pose of the CRBN-BRD4 complex and the corresponding binding moieties to design the PROTACs linker. The process constrains the network to learn from a particular binding pocket corresponding to the released crystal pose in the PDB. Furthermore, the study focuses on a single PROTACs target for testing and validating the proposed model. In the next study, we aim to expand the utilization of the model and apply it to more PROTACs targets and substantially investigate the applications in other structure-based drug discovery.

## Data Availability

The source code that supports the findings of this study is available upon reasonable request from the authors. All data mentioned in this study are publicly available at the ZINC data set, PROTAC-DB, and PDB. We retrieved the training and validation data from the above databanks. All the data we applied can be found in the Supporting Information and at https://github.com/AnHorn/AIMLinker.

## Supporting Information Available

Details on the neural network implementation, benchmark comparison to other methods, and data preparation. AIMLinker hyperparameter tuning in Table S1; performance on filtering generated molecules by using other ML methods in Table S2; structures of postprocess filters in Figure S1; truncated side chain of 3DLinker input fragments in Figure S2 (ZIP)
