# Supplementary figures and images for: Fragment Linker Prediction Using the Deep Encoder-Decoder Network for PROTACs Drug Design
Source: J Chem Inf Model. 2023 May 8;63(10):2918–27. doi: 10.1021/acs.jcim.2c01287 (PMC10207268; doi:10.1021/acs.jcim.2c01287)

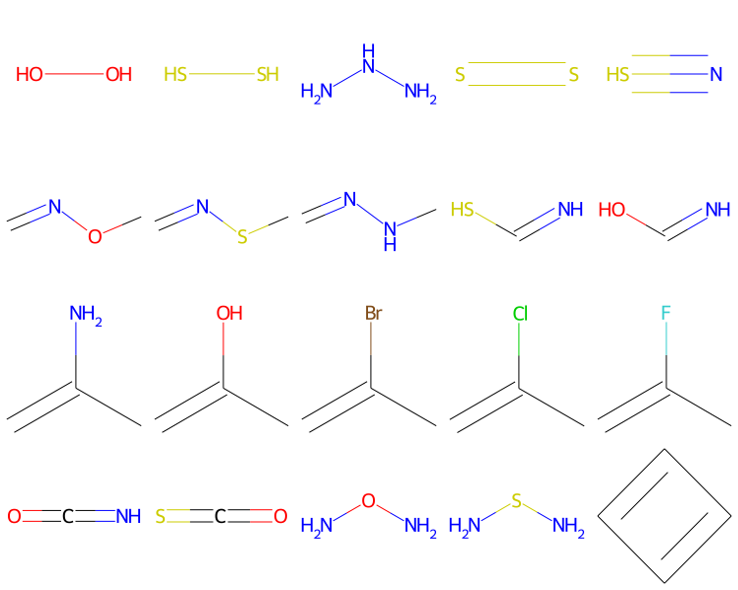

Supplement: Supplementary file 1 — ci2c01287_si_001.zip [file ci2c01287_si_001.zip › figures/filter1.png]

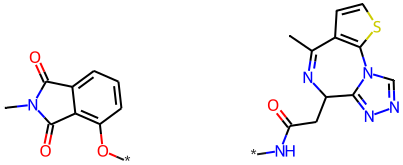

Supplement: Supplementary file 1 — ci2c01287_si_001.zip [file ci2c01287_si_001.zip › figures/3DLinker.png]
